# Serum Thioredoxin-80 is associated with age, ApoE4, and neuropathological biomarkers in Alzheimer’s disease: a potential early sign of AD

**DOI:** 10.1186/s13195-022-00979-9

**Published:** 2022-02-24

**Authors:** Julen Goikolea, Gorka Gerenu, Makrina Daniilidou, Francesca Mangialasche, Patrizia Mecocci, Tiia Ngandu, Juha Rinne, Alina Solomon, Miia Kivipelto, Angel Cedazo-Minguez, Anna Sandebring-Matton, Silvia Maioli

**Affiliations:** 1 Department of Neurobiology, Care Sciences and Society, Division of Neurogeriatrics, Center for Alzheimer Research, Karolinska Institutet, Stockholm, Sweden; 2 Biodonostia Health Research Institute, Neuroscience Area, 20014 Donostia-San Sebastián, Gipuzkoa Spain; 3 CIBERNED (Network Center for Biomedical Research in Neurodegenerative Diseases), Carlos III Institute, Madrid, Spain; 4 Department of Physiology, Medicine and Nursing School, University of Basque Country UPV/EHU, Leioa, Spain; 5 Department of Neurobiology, Care Sciences and Society, Division of Clinical Geriatrics, Center for Alzheimer Research, Karolinska Institutet, Stockholm, Sweden; 6 Department of Medicine and Surgery, Santa Maria della Misericordia Hospital, Section of Gerontology and Geriatrics, University of Perugia, Perugia, Italy; 7 Department of Public Health Solutions, Public Health Promotion Unit, Finnish Institute for Health and Welfare, Helsinki, Finland; 8 Turku PET Centre, University of Turku and Turku University Hospital, Turku, Finland; 9 Institute of Clinical Medicine/Neurology, University of Eastern Finland, Kuopio, Finland; 10 Ageing Epidemiology (AGE) Research Unit, School of Public Health, Imperial College London, London, UK; 11 Institute of Public Health and Clinical Nutrition, University of Eastern Finland, Kuopio, Finland; 12 Theme Aging, Karolinska University Hospital, Stockholm, Sweden

**Keywords:** Thioredoxin-80, Inflammation, Dementia, ApoE4, Alzheimer’s disease, Aging

## Abstract

**Background:**

Thioredoxin-80 (Trx80) is a cleavage product from the redox-active protein Thioredoxin-1 and has been previously described as a pro-inflammatory cytokine secreted by immune cells. Previous studies in our group reported that Trx80 levels are depleted in Alzheimer’s disease (AD) brains. However, no studies so far have investigated peripheral Trx80 levels in the context of AD pathology and whether could be associated with the main known AD risk factors and biomarkers.

**Methods:**

Trx80 was measured in serum samples from participants from two different cohorts: the observational memory clinic biobank (GEDOC) (*N* = 99) with AD CSF biomarker data was available and the population-based lifestyle multidomain intervention trial Finnish Geriatric Intervention Study to Prevent Cognitive Impairment and Disability (FINGER) (*N* = 47), with neuroimaging data and blood markers of inflammation available. The GEDOC cohort consists of participants diagnosed with subjective cognitive impairment (SCI), mild cognitive impairment (MCI), and AD, whereas the FINGER participants are older adults at-risk of dementia, but without substantial cognitive impairment. One-way ANOVA and multiple comparison tests were used to assess the levels of Trx80 between groups. Linear regression models were used to explore associations of Trx80 with cognition, AD CSF biomarkers (Aβ42, t-tau, p-tau and p-tau/t-tau ratio), inflammatory cytokines, and neuroimaging markers.

**Results:**

In the GEDOC cohort, Trx80 was associated to p-tau/t-tau ratio in the MCI group. In the FINGER cohort, serum Trx80 levels correlated with lower hippocampal volume and higher pro-inflammatory cytokine levels. In both GEDOC and FINGER cohorts, ApoE4 carriers had significantly higher serum Trx80 levels compared to non-ApoE4 carriers. However, Trx80 levels in the brain were further decreased in AD patients with ApoE4 genotype.

**Conclusion:**

We report that serum Trx80 levels are associated to AD disease stage as well as to several risk factors for AD such as age and ApoE4 genotype, which suggests that Trx80 could have potential as serum AD biomarker. Increased serum Trx80 and decreased brain Trx80 levels was particularly seen in ApoE4 carriers. Whether this could contribute to the mechanism by which ApoE4 show increased vulnerability to develop AD would need to be further investigated.

**Trial registration:**

ClinicalTrials.govNCT01041989. Registered on 4 January 2010—retrospectively registered

**Supplementary Information:**

The online version contains supplementary material available at 10.1186/s13195-022-00979-9.

## Introduction

Alzheimer’s disease (AD) is the leading cause of dementia and it is considered a multifactorial and complex disorder [1]. The main pathological hallmarks in AD brains are neurofibrillary tangles (NFT) composed of hyperphosphorylated tau protein (p-tau) and amyloid plaques consisting of aggregated amyloid beta (Aβ). It has been proposed that these events, in turn, may lead to neuroinflammatory processes led by microglia, synaptic and neuronal dysfunction, and ultimately brain atrophy [2]. The vast majority of AD cases occur sporadically, known as late-onset AD (LOAD), and are driven by a complex combination of genetics and environmental factors [1].

While aging is still the highest risk factor for AD, several susceptibility genes have been reported [3]. Among them, apolipoprotein E4 (ApoE4) is regarded as the strongest genetic risk factor for LOAD [4]. ApoE has three isoforms (ε2, ε3 and ε4) where the ApoE4 genotype confers higher risk for AD as compared to ApoE3 and ApoE2. Carrying one copy of *APOE4* increases AD risk by 3–4 fold and two copies by 10–15 fold compared to those carrying two copies of *APOE3* [5–7]. The ApoE4 isoform has a decreased capacity to bind and transport lipids including cholesterol affecting lipid homeostasis [8], yet its mechanisms of action involve also other disease pathways. Previous studies show that the presence of the ApoE4 isoform accelerates Aβ accumulation, gliosis, and tau phosphorylation compared with the other isoforms [9, 10]. ApoE4 has also been shown to increase inflammation in mouse brain [11]. Indeed, mice expressing humanized ApoE4 and administered with LPS showed a significant increase in TNFα and IL-6 in the brain in comparison to ApoE3 mice [4]. In the periphery, APOE4 affects the oxidative status in macrophages, producing more superoxide anion radicals than ApoE3 macrophages [12]. Altogether, ApoE4 seems to have an impact on redox and inflammatory processes, two central events in AD.

Thioredoxin-1 (Trx1) is a highly conserved endogenous dithiol with many different roles, including reactive oxygen species (ROS) scavenging to chemokine activities, and it is decreased in AD neurons [13]. Trx1 can be cleaved by the α-secretase activity of ADAM10/17 into an 80 amino-acid long peptide, known as Thioredoxin-80 (Trx80) [14]. Most of the studies regarding Trx80 function have been performed in peripheral blood mononuclear cells where it triggers innate immunity by inducing the activation and differentiation of human monocytes [15], the upregulation of cell surface pathogen recognition receptors, and the production of several pro-inflammatory cytokines [16]. Previous studies reported that Trx80 levels increase significantly in serum with aging and under chronic inflammatory conditions [17–19]. Our lab showed that Trx80 levels are depleted in brains and cerebrospinal fluid (CSF) of MCI patients who later converted to AD [14], suggesting a potential use of Trx80 as a biomarker of relevance for AD progression. However, no studies so far have investigated peripheral Trx80 levels in the context of neurodegeneration and AD.

In the current exploratory study, Trx80 was measured in serum samples from subgroups of two different cohorts: a observational memory clinic cohort GEDOC and the population-based cohort from the lifestyle intervention trial Finnish Geriatric Intervention Study to Prevent Cognitive Impairment and Disability (FINGER). The GEDOC group (*n* = 99) contains patients diagnosed with subjective cognitive impairment (SCI), mild cognitive impairment (MCI), and AD, whereas the FINGER RCT included participants from the general population at risk of dementia but without substantial cognitive impairment (*n* = 47).

The aim of this study was to investigate (a) whether serum Trx80 levels are altered at different stages of dementia and (b) whether serum Trx80 levels are associated with demographical or clinical AD risk factors. Additionally, we aimed to investigate whether any found associations to Trx80 could also be found in a subgroup of at-risk participants of FINGER where we analyzed Trx80 associations with dementia-related markers. Finally, we measured Trx80 levels in AD postmortem brain tissue of ApoE3 and ApoE4 patients.

## Materials and methods

### GEDOC memory clinic population

This study included 99 patients equally distributed between the clinical diagnostic groups subjective cognitive impairment (SCI) mild cognitive impairment (MCI) and AD from the Karolinska University Hospital memory clinic in Huddinge, Sweden. The demographical characteristics of the GEDOC memory clinic population are described in Table 1. The clinical and demographical data relevant for the study (e.g., ApoE genotype) was acquired from the central GEDOC database (Karolinska University Hospital, Stockholm, Sweden).

**Table 1:** Demographic, clinical, and biomarker data of GEDOC memory clinic cohort

	***N***	SCI, mean (SD)	***N***	MCI, mean (SD)	***N***	AD, mean (SD)	***p***
**Demographic data**							
Sex, % men/women	8/17	32.00/68.00	10/14	41.67/58.33	8/17	32.00/68.00	0.72
Age, years	25	59.00 (7.85)	24	69.21 (6.69)	25	73.40 (10.48)	**0.0001**
Years of education	25	14.28 (2.79)	24	11.75 (3.65)	25	10.32 (3.08)	**0.001**
**Cognition**							
MMSE test score	25	29.24 (1.16)	23	27.30 (1.84)	25	24.52 (3.63)	**0.0001**
**ApoE genotype**							
ApoE4 carrier, % yes/no	6/9	40.00/60.00	10/12	45.45/54.55	6/8	42.86/57.14	0.95
ApoE allele frequencies, %							
e2/e2	1	2.70	0	0.00	0	0.00	
e2/e3	2	5.41	2	6.45	2	6.45	
e2/e4	1	2.70	2	6.45	0	0.00	
e3/e3	6	16.22	10	32.26	6	19.35	
e3/e4	4	10.81	6	19.35	5	16.13	
e4/e4	1	2.70	2	6.45	1	3.23	
Unknown	22	59.46	9	29.03	17	54.84	
**CSF measurements**							
Aβ42, ng/l	37	935.32 (229.86)	31	881.87 (350.37)	31	658.32 (302.59)	**0.011**
t-Tau , ng/l	37	224.97 (91.82)	31	325.58 (162.98)	31	499.71 (228.49)	**0.001**
p-Tau, ng/l	37	46.46 (15.32)	31	59.13 (25.95)	31	81.03 (28.17)	**0.01**
**Serum measurements**							
Trx80, ng/ml	37	3.56 (4.87)	31	28.13 (30.39)	31	25.50 (28.58)	**0.0001**

Values are means ± SD unless otherwise specified. Between-group differences were analyzed with chi-square and ANOVA as appropriate. Cognitive scores are mean values, where higher scores indicate better performance. ANCOVA tests adjusting for age were used to compare CSF biomarker and Trx80 levels between groups. *p* value was considered significant (marked in bold) if < 0.05

*ApoE4* apolipoprotein E4, *SCI* subjective cognitive impairment, *MCI* mild cognitive impairment, *AD* Alzheimer’s disease, *CSF* cerebrospinal fluid, *A*β*42* amyloid-beta 42 fragment, *t-Tau* total tau protein, *p-Tau* phosphorylated tau protein, *MMSE* mini mental state examination

Diagnosis for MCI was done using the consensus criteria for MCI which require the presence of both subjective and objective cognitive impairment including one or several cognitive domains, but no dementia or impairment of daily living activities [20]. Dementia diagnoses were carried out following the criteria of the Diagnostic and Statistical Manual of Mental Disorders, 4th edition (DSM-IV), as previously described [21].

Routine neurological and physical examinations were carried out at the memory clinic as described previously [21] and included Mini-Mental State Examination (MMSE), blood tests and CSF sampling. CSF samples were collected as previously described [21]. Fresh samples were used to measure soluble Aβ42, t-tau, and p-tau concentrations in CSF with commercially available sandwich enzyme-linked immunosorbent assays (ELISA) (Innogenetics, Belgium) according to standardized protocols in the memory clinic.

### FINGER study participants

**Fig. 1:**
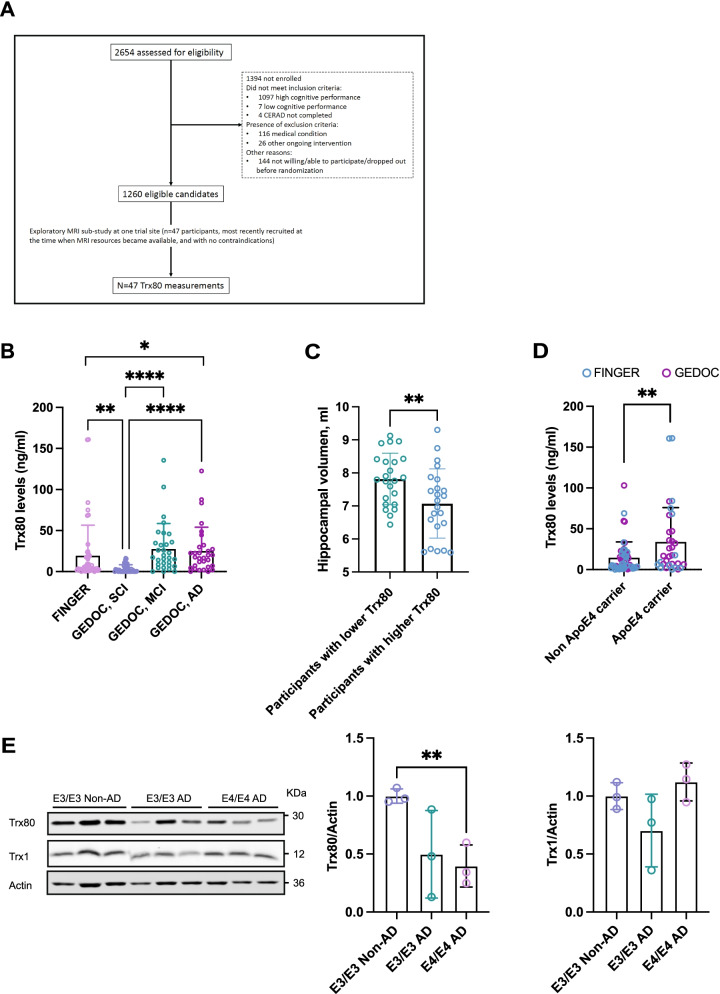
**A** CONSORT diagram of the FINGER exploratory Thioredoxin-80 sub-study. CERAD, Consortium to Establish a Registry for Alzheimer’s Disease. **B** Serum Trx80 levels by disease diagnosis. Participants were divided into four groups according to their disease state. FINGER cohort participants and SCI, MCI, and AD participants from the GEDOC cohort. The graph shows serum Trx80 levels (ng/ml) between groups. *p* values are calculated from one-way ANCOVA adjusted for age. **C** Hippocampal volume of patients with highest and lowest serum Trx80 levels in FINGER cohort. The graph shows hippocampal volume (ml) between groups. *t*-test was used to analyze the differences between groups. **D** Serum Trx80 levels in ApoE4 carriers and non-carriers from merged GEDOC and FINGER cohorts. The graph shows serum Trx80 levels (ng/ml) between groups. *p* values are calculated from one-way ANCOVA adjusted for age. **E** Western-bot analysis of Trx80 levels in post-mortem AD and non-AD brain samples. Samples are sorted by APOE genotype ApoE3/ApoE3 (E3/E3) and E4/E4. Student *t*-test was used to analyze the differences between groups. *p < 0.05; **p < 0.01; ****p < 0.0001

This exploratory sub-study included baseline data from 47 FINGER trial participants (21 women and 26 men, mean age 71 ± 5.1 years) who underwent neuroimaging in Turku (Finland) [22] with serum samples available for Trx80 measurements (For CONSORT flowchart, see Fig. 1A). They were selected at the time when MRI resources became available, and if there were no contraindications. The demographical and clinical characteristics of these participants have been previously described [22, 23], and they were not different from the rest of the FINGER participants.

The FINGER population characteristics [24], trial protocol details [25], main relevant findings [26], and neuroimaging sub-study have been previously published in detail [22, 27]. The demographical, clinical, and neuroimaging data relevant for the study was acquired from the central FINGER database (Finnish Institute for Health and Welfare, Helsinki, Finland). In brief, the data collection was ongoing between September 7, 2009, and November 24, 2011, where 2654 individuals were screened, and 1260 participants of ages between 60 and 77 years from the general population were recruited based on cognitive performance at the mean level or slightly lower than expected for their age according to Finnish population norms for the Consortium to Establish a Registry for Alzheimer’s Disease (CERAD) [28] and 6 points or higher in the Cardiovascular Risk Factors, Aging and Dementia (CAIDE) risk score [29]. Diagnosis of dementia or substantial cognitive impairment was used as reason for exclusion as well as any condition affecting safe participation.

### Ethics approval

The study was approved by the Regional Ethical Review Board in Stockholm, and written informed consent was obtained from all participants.

### Cognitive outcomes

Mini-Mental State Examination (MMSE) was conducted as standard cognitive testing for all GEDOC and FINGER participants. Higher scores indicated better performance.

### MRI imaging

All participants underwent a brain 3T MRI (3D Turbo Field Echo sequence, voxel size 1.0 × 1.0 × 1.0 mm, total slices 160, field of view 240 × 240 mm, repetition time 8.1 ms, echo time 3.7 ms, Philips Ingenuity TF PET/MR, Amsterdam, the Netherlands). The specific protocol for MRI imaging has been previously described [22]. T1WI and FLAIR images were quality checked and visually inspected for any abnormalities by an experienced neuroradiologist. Images were excluded if there were brain lesions potentially affecting volumetry and/or scanning issues. Regular phantom scans were performed, and quantitative measures of signal-to-noise ratio, uniformity, and geometric distortion were carried out.

Freesurfer image analysis (version 5.3.0) was used to measure cortical thickness and regional brain volumes. Manual editing was performed in cases where automated white matter (WM) segmentation presented geometric inaccuracies in boundaries between CSF, gray, and white matter. Brain volumes were adjusted for head size to account for between-patient variations in head size [30]. Cortical thickness measurement in AD signature regions was calculated as the mean average of cortical thickness in the entorhinal, inferior temporal, middle temporal, and fusiform regions [31].

White matter lesions (WML) volume was measured through the segmentation of WM hyperintensities on T1 and FLAIR images according to a previously described method [32]. The segmentation was done in 3 steps based on the expectation-maximization algorithm: [1] from the T1 images, WM was segmented into two classes, normal-bright WM regions, and hypointense WM regions [2]; FLAIR images were segmented to three classes: CSF, normal brain tissue and hyperintense voxels [3]; the WM and subcortical regions were segmented into two classes from the FLAIR images. The segmentation of WM hyperintensities was regarded as the class with higher intensities [32, 33].

### Thioredoxin-80 ELISA measurements

Determination of Trx80 levels in serum was performed with a sandwich ELISA as previously described [34] but with a few modifications. In brief, standard samples of recombinant human Trx80 (#11522), coating anti-Trx80 monoclonal mouse antibody (clone 7D11 #11543), and detection antibody (biotinylated goat polyclonal anti-Trx1 #11541) were purchased from Cayman chemicals (USA). Standard dilutions of Trx80 (0.2–125 ng/ml) and serum samples were prepared in blocking buffer (0.5% BSA, 0.05% Tween-20); 50 μl of standards or samples were added in duplicates and incubated overnight (O/N). Biotinylated goat anti-Trx1 antibody was prepared at a concentration of 2 μg/ml in blocking buffer (50 μl/well) and incubated 2 h at room temperature. Streptavidin-HRP (#OR03L, Merck, USA) was diluted to 10 ng/ml in PBS and incubated 45 min at room temperature. TMB substrates (#34021, Thermo Fisher Scientific, USA) were mixed 1:1, added 100 μl/well, and incubated 25 min in the dark; 50 μl/well of stop solution (2N sulfuric acid, DY994, R&D systems, USA) was added and absorbance was immediately measured at 450 nm in the spectrophotometer (Tecan Safire 2 Multi-Detection Plate Reader, Switzerland).

### Plasma cytokines and chemokines measurements in FINGER

In the FINGER study, venous blood samples were taken at baseline in fasting status and using EDTA tubes. Plasma aliquots were stored at – 80 °C until analysis. A panel of cytokines, chemokines, and growth factors (IL-1α, IL-1β, IL-2, IL-3, IL-4, IL-5, IL-6, IL-7, IL-8, IL-10, IL-12(p40), IL-12(p70), IL-13, IL-15, IL-17, IFN-α2, IFN-γ, MIP-1α, TNF-β) were analyzed with a multiplex suspension array system using Bioplex Luminex 200 instrument (Bio-Rad Laboratories, Hercules, CA, USA) and the MILLIPLEX® MAP Human Cytokine/Chemokine panel (Merck Millipore, Darmstadt, Germany).

The assays were performed in one batch, and samples preparation and setting of the system running protocol were done following the manufacturer’s instructions. All samples and standards were run in duplicate and were measured as pg/ml. Quality controls were performed according to the manufacturer guidelines to ensure accuracy of measurements. After the plate reading, the results files were generated using Bio-Plex Manager software 4 (Bio-Rad Laboratories, Hercules, CA, USA).

### Human brain samples

Human brain tissue samples were obtained from CIEN foundation tissue bank for neurological research (BT-CIIN). Information regarding age, sex, ApoE4 genotype, postmortem interval, and dementia diagnosis from the brain tissue is displayed in Supplementary Table 1.

### Sample preparation and western blot

Immunoblotting was performed as described [35]. Briefly, human brain tissue was homogenized in lysis buffer (50 mM Tris-HCL, 150 mM NaCl, 1% Triton-X) containing phosphatase and protease inhibitors (Sigma-Aldrich, USA). Homogenates were then centrifuged at 15000×g for 15 min 4 °C, and samples were mixed with equal volumes of loading buffer (160 mM Tris-HCl pH 6.8, 4% SDS, 20% glycerol, 0.01% bromophenol blue, 100 mM DTT). Processed samples were then run in 12.5% polyacrylamide gels (Bio-Rad, USA) and transferred to BioTraceTM nitrocellulose membranes (GE healthcare, USA) for 2 h room temperature at 50 V. Membranes were then blocked for 1 h in 5% skim milk in TBS-Tween-20 (TBS-T) prior to overnight incubation with diluted primary antibody at 4 °C. Primary antibodies: 1:1000 anti-Trx80 monoclonal mouse antibody (clone 7D11 #11543, Cayman chemicals, USA), 1:1000 anti-Trx1(human) Polyclonal Goat Antibody (#11538 Cayman chemicals, USA), and 1:10000 monoclonal Anti-β-Actin mouse antibody, clone AC-15 (Sigma-Aldrich, USA). Fluorescent secondary antibodies (1:10000, LI-COR Biosciences, USA) were used for 2 h at room temperature, and bands were visualized using ODYSSEY Infrared Imaging System (LI-COR Biosciences). Band intensity signal was quantified by ImageJ software. Each band signal value was normalized against loading control (actin signal value).

### Statistical analysis

Demographic data were compared across clinical diagnostic groups with ANOVA for continuous and *χ*^2^ for categorical variables. Simple regression analysis was applied when exploring the relationship between age, sex and ApoE4 carriership and Trx80 levels. Further associations were analyzed by multiple linear regression models adjusted for age and clinical diagnosis when appropriate. For variables that were not normally distributed (CSF AD biomarkers (Ab42, t-tau, p-tau and p-tau/t-tau ratio), inflammatory cytokines and Trx80), zero-skewness log transformation was applied. ANCOVA tests adjusting for age were used to compare CSF biomarker and Trx80 levels between groups and Trx80 levels between ApoE4 carriers and non-carriers. Student *t*-test was used to compare hippocampal volumes between groups. Correction for multiple testing has not been applied, and all the analyses performed are post hoc. Level of significance was set to *p* < 0.05 in all analyses. Analysis was performed using the Stata software, version 14 (StataCorp), and GraphPad Prism, version 9 (GraphPad Software, CA).

## Results

### Population characteristics of GEDOC and FINGER sub-studies

Demographical and clinical data as well as CSF and serum measurements from a subset of the Karolinska University Hospital memory clinic cohort GEDOC are listed in Table 1. The MCI and AD groups were significantly older than the SCI group (69 ± 7 and 73 ± 10 versus 59 ± 8 years, *p* < 0.0001), had lower level of education (12 ± 4 , 10 ± 3 versus 14 ± 3 years, *p* < 0.001), and had lower MMSE scores compared to SCI (27.30 ± 1.84, 24.52 ± 3.63 versus 29.24 ± 1.16 points, *p* < 0.0001). As expected from previous reported measurements [2], both MCI and AD groups had significantly lower Aβ42 levels compared to SCI (881.87 ± 350.37 ng/l and 658.32 ± 302.59 versus 935.32 ± 229.86 ng/l, *p* < 0.001), higher t-tau levels than the SCI group (324.58 ± 162.98, 499.71 ± 228.49, versus 224.97 ± 91.82 ng/l, *p* < 0.0001), and higher p-tau levels in CSF than SCI group (59.13 ± 25.95, 81.03 ± 28.17 versus 46.46 ± 15.32 ng/l, *p* < 0.0001).

The baseline characteristics of the FINGER sub-study (*n* = 47) are listed in Table 2. In addition to serum Trx80 levels, the herein analyzed data contains demographics, cognitive measurements, distribution of ApoE genotype, neuroimaging, and inflammatory marker data. Since only baseline data were included in this exploratory study, both control and intervention groups are analyzed as one “at risk of dementia” category.

**Table 2:** Demographic, clinical, and biomarker data of the FINGER neuroimaging cohort at baseline

	Number	Mean (±SD)
**Demographical data**		
Sex, % male/female	26/21	55.32/44.68
Age, years	47	70.66 (5.06)
**Cognitive assessment**		
MMSE	47	27.02 (1.78)
**ApoE genotype**		
ApoE4 carriers, % (yes/no)	14/32	30.43/69.57
ApoE allele frequencies (%)		
2.3	2	4.35
2.4	2	4.35
3.3	30	65.22
3.4	11	23.91
4.4	1	2.17
**MRI**		
*Total gray matter volume, ml	47	575.76 (55.50)
*Total hippocampal volume, ml	47	7.47 (0.99)
AD signature cortical thickness, mm	47	2.73 (0.13)
*White matter lesion volume, ml	46	13.27 (16.18)
Total intracranial volume, ml	47	1548.82 (252.08)
**Serum measurements**		
Trx80, ng/ml	47	19.46 (36.37)
**Inflammatory markers (pg/ml)**		
IL-1α	41	1465.39 (2558.60)
IL-1β	41	123.47 (379.81)
IL-2	41	65.23 (159.62)
IL-3	32	15.93 (9.48)
IL-4	41	169.00 (239.26)
IL-5	41	57.16 (78.22)
IL-6	41	58.17 (57.09)
IL-7	41	44.73 (22.54)
IL-8	41	58.17 (57.09)
IL-10	41	151.23 (165.27)
IL-12 p40	41	435.83 (639.53)
IL-12 p70	41	130.07 (149.32)
IL-13	41	112.00 (157.59)
IL-15	41	81.81 (85.99)
IL-17	41	77.17 (104.78)
Interferon-α2	41	204.32 (149.36)
Interferon-γ	41	132.48 (178.42)
Mip-1a	36	43.22 (37.77)
TNF-β	41	109.24 (146.66)
Bradykinin	47	6610000 (3870000)

Values are means ± standard deviation (SD) unless otherwise specified. Cognitive scores are mean values of cognitive tests, where higher scores indicate better performance. *Brain volumes are shown unadjusted; AD signature cortical thickness was calculated as the average of cortical thickness in the entorhinal, inferior temporal, middle temporal, and fusiform regions. *N,* number of participants with available data for each analysis. *MRI* magnetic resonance imaging; *IL-1* interleukin-1, *Mip-1a* macrophage inflammatory protein 1a, *TNF-*b tumor necrosis factor-beta

### Serum Trx80 levels are significantly increased in AD irrespectively of age

As reported in Table 1, the SCI group in GEDOC was significantly younger than MCI and AD groups. According to what previously has been shown [17], age (*β* = 0.33, *p* = 0.00) but not sex (men, 13.33 ± 3.40 ng/ml; women, 19.41 ± 3.44 ng/ml; *p* = 0.26) had a significant influence on Trx80 in the GEDOC cohort. In contrast to the GEDOC cohort, where there was a significant difference in age among participants, in the FINGER cohort, there was no association between serum Trx80 levels and age (*β* = − 0.04, *p* = 0.77) nor with sex (men, 21.26 ± 8.95 ng/ml; women, 18.24 ± 5.41 ng/ml; *p* = 0.26). Based on this, we decided to include FINGER sub-study participants in the comparison since they represent non-demented subjects at risk for AD, whose average age (70 ± 5 years) is comparable to MCI and AD groups. When serum Trx80 levels were analyzed between these 4 groups (Fig. 1B), we observed that the SCI group had significantly lower Trx80 levels (3.56 ± 4.87 ng/ml) than the FINGER sub-study (19.46 ± 38.37 ng/ml; *p* < 0.01), MCI group (28.13 ± 30.39 ng/ml; *p* < 0.001), and AD group (25.50 ± 28.58 ng/ml; *p* < 0.001). When comparing the age matched FINGER sub-study participants to AD, significantly higher Trx80 levels were seen in AD (*p* < 0.02), and a similar trend could be observed for MCI group (*p* < 0.10). Based on this result, we combined the data from both sub-studies (GEDOC and FINGER) to perform further analysis on serum Trx80 associations (*n* = 145).

### Trx80 is associated with age and ApoE4 in GEDOC and FINGER combined dataset

In agreement with the above results, Trx80 levels significantly correlated with age (*β* = 0.23, *p* = 0.01) but not with sex (*β* = 0.14, *p* = 0.12) in the merged group (Table 3). When adjusting for age, there was a significant association between Trx80 levels and ApoE4 carriers (*β* = 0.34, *p* = 0.00).

**Table 3:** Serum Trx80 associations with age, sex, and ApoE4 genotype in a combined dataset

	Non-adjusted ***β*** (***p***)	Adjusted for age ***β*** (***p***)
**Demographic characteristics**		
Age	**0.23 (0.01)**	
Sex	0.14 (0.12)	0.13 (0.14)
ApoE4 carrier	0.17 (0.10)	**0.34 (0.00)**

Values are standardized *β* coefficients (*p* values) from linear regression models with serum Trx80 levels as a dependent variable. Linear regression models are non-adjusted or adjusted for age. *p* values considered significant (bold) if *p* < 0.05

### Serum Trx80 levels are negatively associated with p-tau in the SCI group and positively with p-tau/t-tau ratio in MCI group of the GEDOC cohort

Data on clinical CSF AD biomarkers were available from the GEDOC memory clinic cohort. To explore how Trx80 varies with known AD pathology, we investigated the association between serum Trx80 levels and CSF Aβ42, t-tau, p-tau levels, and p-tau/t-tau ratio in the GEDOC cohort (Table 4). No associations between Trx80 and Aβ42 or Trx80 and tau were found when adjusting for age. As shown in Table 1, serum Trx80 levels are significantly higher in MCI and AD groups in comparison to SCI. Since serum Trx80 is increased with dementia and taking into account that Aβ42, t-tau, and p-tau levels are determinant in the development of AD, we next analyzed whether these markers correlated with serum Trx80 levels at different disease stages when adjusted for age (SCI, MCI and AD, Table 4). Higher Tx80 levels were significantly associated with lower p-tau levels (*β* = − 0.48, *p* = 0.02) in the SCI group, and a similar trend could be observed for t-tau (*β* = − 0.36, *p* = 0.08). In the MCI group, higher p-tau/t-tau ratio was associated with higher Trx80 levels (*β* = 0.41, *p* = 0.05). There were no further significant associations for Aβ42, t-tau, and p-tau or the ratio in MCI or AD groups.

**Table 4:** Associations between serum Trx80 and CSF AD biomarkers in the GEDOC cohort

	Total		SCI		MCI		6AD	
	***N***	***β*** (***p***)	***N***	***β*** (***p***)	***N***	***β*** (***p***)	***N***	***β*** (***p***)
Aβ42	99	− 0.14 (0.23)	37	0.21 (0.38)	31	− 0.15 (0.50)	31	− 0.12 (0.56)
t-tau	99	0.12 (0.31)	37	− 0.36 (0.08)^#^	31	− 0.23 (0.29)	31	0.29 (0.17)
p-tau	99	0.13 (0.27)	37	− **0.48 (0.02)**	31	− 0.03 (0.89)	31	0.27 (0.20)
p-tau/t-tau	99	− 0.07 (0.55)	37	− 0.07 (0.73)	31	**0.41 (0.05)**	31	− 0.21 (0.34)

Values are standardized *β* coefficients (*p* values) from linear regression models with serum Trx80 levels as a dependent variable. Linear regressions are adjusted for age. *p* values considered significant (bold) if *p* < 0.05; #, *p* < 0.10. *N*, number of participants with available data for each analysis

### Serum Trx80 is associated with lower hippocampal volume and higher pro-inflammatory cytokine levels in the FINGER cohort

We next sought to determine if Trx80 is related to brain volume measures. We found that there was a negative association between hippocampal volume and serum Trx80 levels (*β* = − 0.32, *p* = 0.01, Table 5). No other association was found significant between gray matter volume, cortical thickness, or white matter lesions and serum Trx80 levels.

**Table 5:** Associations between serum Trx80 and neuroimaging and inflammatory markers in the FINGER cohort

	Adjusted for age
	***β*** (***p***)
MRI*	
Total gray matter volume, ml	− 0.07 (0.67)
Hippocampal volume, ml	− **0.32 (0.01)**
AD signature cortical thickness, mm	− 0.08 (0.61)
White matter lesions, ml	− 0.11 (0.50)
**Inflammatory markers**	
IL-1α	0.09 (0.59)
IL-1β	0.12 (0.47)
IL-2	0.10 (0.55)
IL-3	0.19 (0.36)
IL-4	0.32 (0.07)
IL-5	0.31 (0.08)
IL-6	0.30 (0.08)
IL-7	0.12 (0.49)
IL-8	**0.33 (0.05)**
IL-10	0.16 (0.33)
IL-12 p40	0.10 (0.56)
IL-12 p70	0.05 (0.77)
IL-13	**0.33 (0.05)**
IL-15	0.14 (0.41)
IL-17	0.17 (0.32)
Interferon-α2	0.29 (0.10)
Interferon-γ	0.28 (0.10)
Mip-1a	**0.40 (0.03)**
TNF-β	0.30 (0.08)
Bradykinin	**0.32 (0.04)**

Values are standardized *β* coefficients (*p* values) from linear regression models with serum Trx80 levels as a dependent variable. Linear regressions are adjusted for age. *Brain volumes are shown adjusted to head-size and time between blood sample collection and brain scan; AD signature cortical thickness was calculated as the average of cortical thickness in the entorhinal, inferior temporal, middle temporal, and fusiform regions. *p* values considered significant (bold) if *p* < 0.05. *MRI* magnetic resonance imaging, *IL-1* interleukin-1, *Mip-1a* macrophage inflammatory protein 1a, *TNF-α* tumor necrosis factor-beta

No clinical cutoff for Trx80 has been reported; however, we wanted to explore if there were differences in neuroimaging data among FINGER participants when they are divided into two groups according to their serum Trx80 levels. Participants with the highest serum Trx80 levels had significantly lower hippocampal volume than the participants with the lowest serum Trx80 levels (7.07 ± 1.05 ml; 7.82 ± 0.78 ml, respectively, *p* < 0.01; Fig. 1C). No differences were found when comparing gray matter volume, cortical thickness or white matter lesions between participants with the highest and lowest Trx80 levels.

Since previous studies reported that Trx80 is implicated in the inflammatory response present in atherosclerotic lesions [17, 19], and it induces a pro-inflammatory response in monocytes and macrophages [15, 36, 37], we investigated possible associations between serum Trx80 levels and serum inflammatory cytokine levels of the participants. Higher Trx80 levels were associated with higher levels of IL-8 (*β* = 0.33, *p* = 0.05), IL-13 (*β* = 0.33, *p* = 0.05), Mip-1a (*β* = 0.39, *p* = 0.03), and bradykinin (*β* = 0.32, *p* = 0.04), and a similar trend, however not significant, was observed for IL-4 (*β* = 0.32, *p* = 0.06), IL-5 (*β* = 0.31, *p* = 0.07), IL-6 (*β* = 0.30, *p* = 0.07), interferon-α (*β* = 0.29, *p* = 0.08), interferon-γ (*β* = 0.28, *p* = 0.10), and TNF-*β* (β = 0.30, *p* = 0.08).

### ApoE4 genotype impacts serum Trx80 levels and its association to CSF Aβ42

As shown in Fig. 1D, ApoE4 carriers from both GEDOC and FINGER cohorts had significantly higher (approximately twofold) Trx80 levels than non-carriers (30.09 ± 41.00 ng/ml; 13.39 ± 18.88 ng/ml, respectively, *p* < 0.01). Due to the significant role of ApoE4 in AD onset and progression, we investigated the associations between Trx80 and CSF AD biomarkers in ApoE4 carriers and non-carriers (Table 6). Higher serum Trx80 levels were associated with lower Aβ42 in CSF (*β* = − 0.46, *p* = 0.04) in non-ApoE4 carriers when adjusted for age. A similar trend could be observed when adjusting for disease diagnosis (*β* = − 0.36, *p* = 0.07). This association could not be observed in ApoE4 carriers. Total tau and p-tau did not show any significant associations with Trx80 in these groups. The ratio p-tau/t-tau was significantly associated to serum Trx80 levels in ApoE4 non-carriers (*β* = 0.49, *p* = 0.00).

**Table 6:** Serum Trx80 associations with CSF Aβ42, p-tau, and t-tau in ApoE4 carriers and non-carriers

	Adjusted for age				Adjusted for diagnosis			
	ApoE4 non-carriers		ApoE4 carriers		ApoE4 non-carriers		ApoE4 carriers	
	***N***	***β*** **(*p*)**	***N***	***β*** **(*p*)**	***N***	***β*** **(*p*)**	***N***	***β*** **(*p*)**
Aβ42	**29**	**− 0.48 (0.04)**	22	0.51 (0.11)	29	**−** 0.36 (0.07)^#^	22	0.27 (0.25)
t-tau	29	**−** 0.13 (0.60)	22	**−** 0.39 (0.18)	29	**−** 0.38 (0.06)^#^	22	**−** 0.04 (0.81)
p-tau	29	0.04 (0.87)	22	**−** 0.32 (0.27)	29	**−** 0.19 (0.37)	22	**−** 0.09 (0.62)
p-tau/t-tau	29	0.34 (0.16)	22	0.23 (0.50)	**29**	**0.49 (0.00)**	22	0.01 (0.98)

Values are standardized *β* coefficients (*p* values) from linear regression models with serum Trx80 levels as a dependent variable in GEDOC cohort. Linear regressions are adjusted for age and disease diagnosis. Corresponding *p* values are shown from analyses with values adjusted zero-skewness log-transformed as appropriate. *p* values considered significant (bold) if *p* < 0.05; #, *p* < 0.10. *N*, number of participants with available data for each analysis

We also investigated whether the associations between ApoE4 and MMSE scores with Trx80 were affected over the disease spectrum (Table 7). To this end, we analyzed these associations within each cohort (FINGER participants and memory clinic patients (SCI, MCI and AD)). A positive association was found between serum Trx80 levels and ApoE4 in FINGER participants (*β* = 0.30, *p* = 0.05) and in the MCI group from GEDOC cohort (*β* = 0.59, *p* = 0.02). This association was not found in the SCI neither in the AD groups. Regarding cognitive score data, there was a significant association between high Trx80 levels and low MMSE scores only the MCI group (*β* = − 0.41, *p* = 0.05).

**Table 7:** Serum Trx80 associations with ApoE4 and MMSE scores from the combined dataset

	FINGER		SCI		MCI		AD	
	***N***	***β*** (***p***)	***N***	***β*** (***p***)	***N***	***β*** (***p***)	***N***	***β*** (***p***)
ApoE4 carriers (yes/no)	14/32	**0.30 (0.05)**	6/9	0.41 (0.50)	10/12	**0.59 (0.02)**	6/8	− 0.28 (0.55)
MMSE	47	0.21 (0.17)	25	− 0.05 (0.81)	23	− **0.41 (0.05)**	25	− 0.03 (0.87)

Values are standardized *β* coefficients (*p* values) from linear regression models with serum Trx80 levels as a dependent variable in FINGER and GEDOC cohorts. Linear regression models are adjusted for age. In ApoE4 regression models, a positive coefficient is interpreted as Trx80 being higher in ApoE4 carriers. *p* values considered significant (bold) if *p* < 0.05. *N*, number of participants with available data for each analysis

### Trx80 levels are decreased in ApoE4 AD brains

Finally, we measured Trx80 protein levels in post-mortem human brain homogenates of AD and non-AD patients. Demographical and clinical data from the brain samples has been collected in Supplementary Table 1. AD samples were grouped by ApoE genotype in ApoE3/ApoE3 (E3/E3) or E4/E4 (Fig. 1E). Despite the low number sample size (*n* = 3 per group), only E4/E4 AD patients had significantly lower Trx80 levels than E3/E3 non-demented controls (*p* < 0.01). As previously reported by our group [14], Trx80 levels in E3/E3 AD patients were also lower than the non-AD controls, although this difference did not reach statistical significance (*p* = 0.08) in this short sample number study. There were no significant differences in the precursor Trx1 protein levels between groups.

## Discussion

Trx80 has been previously described as a pro-inflammatory cytokine secreted by immune cells in the periphery [15, 34, 36]. Previous studies showed that Trx80 is able to prevent Aβ aggregation and to inhibit Aβ toxic effects in cell cultures and that its levels are reduced in AD brain and CSF [14]. Further studies on *Drosophila melanogaster* models overexpressing human Trx80, human Aβ42, or both Aβ42/Trx80 in the central nervous system developed in our lab showed that Trx80 expression prevented Aβ42 accumulation in the brain [38]. In view of these evidence that link Trx80 with AD pathology and inflammatory responses, in the present study, we investigated the possible associations of serum Trx80 levels to several known AD risk factors and AD biomarkers. We found that serum Trx80 levels significantly increase in AD and are positively associated to old age and ApoE4 genotype, two of the main risk factors for AD. Also, high Trx80 serum levels correlated with lower hippocampal volume in a non-demented but at-risk population to worse cognitive test performance in MCI patients. Finally, we found that in ApoE4 AD postmortem brains, Trx80 levels are consistently lower that in ApoE3 AD brain samples.

Previous reduced Trx80 levels in brain from AD patients [14] were confirmed here in a limited number of samples. This is in apparent discrepancy with the increased Trx80 levels in serum from AD patients seen in the present study. However, one possibility to explain the divergency between Trx80 levels in the brain and in the periphery is that they might represent two independent pools, produced from different sources in the body. Trx80 in the brain, produced by neurons and astrocytes [14], gets progressively depleted as neurodegeneration occurs in AD, likely due to a reduction in the production of Trx80 precursor (Trx1) [39] and/or a downregulation of the enzyme (or enzymes) that cleave Trx1 to Trx80. On the other hand, peripheral Trx80 is produced by innate immune cells such as monocytes and likely to be also produced by other cell types in the body. Thus, peripheral Trx80 production might not get affected by neurodegeneration. Trx80 has the function of an inflammatory cytokine, and its levels in blood have been reported to rise under external infections [15], and during chronic inflammatory processes such as rheumatoid arthritis [18] or atherosclerosis [19]. Thus, it is plausible that serum Trx80 might also increase when chronic inflammatory processes occur in the brain, i.e., AD. Nevertheless, further work needs to be done in order to understand the relationship between peripheral and brain Trx80.

It has been reported that high levels of pro-inflammatory markers in the blood and other tissues are more often detected in older individuals [40] and that they may promote neurodegenerative events that can contribute to AD [41]. In the ARIC study, the relationship between plasma inflammatory markers during midlife and neurodegeneration 20 years later was investigated [42]. It was found that participants with elevated midlife inflammatory markers had smaller hippocampal volume and a faster cognitive decline [43]. In our study, we show that high serum Trx80 levels are associated with higher levels of pro-inflammatory cytokines such as IL-8, IL-13, IL-5, IL-6, interferon-α2, and interferon-γ in FINGER study participants. Moreover, high serum Trx80 levels are associated with a smaller hippocampal volume and lower cognitive tests scores. Another peptide related to inflammatory changes in AD, soluble Trem2 (sTrem2) has also been found increased in CSF samples of MCI and early-AD patients, whereas its levels decrease at more advanced stages of the disease [44]. Thus, Trx80 and sTrem2, both products of ADAM10 cleavage, seem to show a similar pattern in their levels across AD disease stages.

We show that ApoE4 genotype, the major genetic risk factor for AD, is associated to Trx80. Indeed, ApoE4-carriers have higher serum Trx80 levels than non-carriers in both, FINGER participants at risk for AD and memory clinic patients of the GEDOC cohort. Previous studies show that Trx80 levels increase in response to inflammatory stimuli [18]. Given the presence of ApoE4 is a known contributor of several pathogenic pathways in AD [45], including inflammation, it is possible that higher Trx80 levels are being produced as a response to these effects.

Specifically, the association between ApoE4 carriers and serum Trx80 levels was only present in FINGER participants and GEDOC MCI group but not in advanced stages of the disease. Whether this association was masked by the low number of known ApoE4 carriers in the ApoE4-AD group or whether it occurs in early stages of the disease needs to be further investigated. Opposite to what it is observed in serum, Trx80 levels in the brain are further decreased in ApoE4-AD than in ApoE3-AD brain samples. Previous studies show that ADAM10 levels and activity in neurons is significantly reduced in AD patients in comparison to age-matched controls [46–48] and that the presence of ApoE4 reduces ADAM10 activity levels in comparison to other ApoE isoforms [49]. These results could potentially explain the decrease of Trx80 in ApoE4 AD brain samples. As Trx80 shows neuroprotective and anti-amyloid effects in the brain, an increased depletion of Trx80 could contribute to the increased pathogenesis and faster progression of AD in ApoE4 carriers [50, 51]. In serum, lower Trx80 correlates with higher CSF Aβ42 levels (which in turn is associated with lower amyloid-beta deposition in the brain) [2], and non-ApoE4 carriers showed significantly lower Trx80 levels than ApoE4 carriers, supporting the notion of an inverse relationship between brain and serum Trx80 levels that is further apparent AD individuals carrying ApoE4.

### Limitations

The main limitation of this study is the relatively small sample size of our cohorts that affects the statistical power. This is especially a problem when comparing ApoE4 carrier data between groups. Because no correction for multiple testing has been applied and all the analysis are post hoc, the findings presented above should be considered exploratory and will need to be further verified in studies with larger sample sizes.

## Conclusions

Trx80 is a truncated peptide with inflammatory functions in the periphery and anti-amyloidogenic properties in brain. In this study, we report that serum levels of Trx80 peptide are increased in AD patients and are associated with age, ApoE4 genotype, and with p-tau/t-tau ratio in early stages of AD. We found that serum Trx80 levels correlate with lower hippocampal volume and higher pro-inflammatory cytokine levels in individuals at risk of dementia. Importantly, the ApoE4 carriers show higher Trx80 levels in serum and lower in brain as compared to non-ApoE carriers. This effect could be observed in dementia patients as well as patients at-risk but without notable cognitive impairment. Understanding why age and ApoE4 genotype, two main risk factors for AD, affect Trx80 levels in serum and brain in opposite manners might allow a better understanding of early events in AD neurodegeneration. Moreover, further studies with larger number serum and CSF samples are needed to explore if measuring Trx80 could contribute to an earlier detection of AD.

## Supplementary Information

**Additional file 1: Supplementary Table 1**. Demographic and clinical data from the human brain tissue samples.

## Data Availability

The datasets used and/or analyzed during the current study are available from the corresponding author on reasonable request.

## Abbreviations

Aβ: Amyloid beta
AD: Alzheimer’s disease
ApoE4: Apolipoprotein allele E4
ARIC study: Atherosclerosis risk in communities study
BSA: Bovine serum albumin
CAIDE: Cardiovascular Risk Factors, Aging and Dementia
CERAD: Consortium to Establish a Registry for Alzheimer’s Disease
CSF: Cerebrospinal fluid
ELISA: Enzyme-linked immunosorbent assay
FINGER: Finnish Geriatric Intervention Study to Prevent Cognitive Impairment and Disability
GEDOC: Clinical-based database for patients at Karolinska University Hospital memory clinic Huddinge
HRP: Horseradish peroxidase
IL-1: Interleukin-1
MCI: Mild cognitive impairment
Mip-1a: Macrophage inflammatory protein-1a
MMSE: Mini mental state examination
MRI: Magnetic resonance imaging
NINCDS-ADRDA: National Institute of Neurological and Communicative Disorders and Stroke–Alzheimer’s Disease and Related Disorders Association criteria
PIB-PET: Pittsburgh compound B–positron emission tomography
p-tau: Phosphorylated tau
SCI: Subjective cognitive impairment
t-tau: Total tau
Trx1: Thioredoxin-1
Trx80: Thioredoxin-80
TNF-β: Tumor necrosis factor-beta
WML: White matter lesion
