# Supplementary material for: Serum Thioredoxin-80 is associated with age, ApoE4, and neuropathological biomarkers in Alzheimer’s disease: a potential early sign of AD
Source: Alzheimers Res Ther. 2022 Feb 24;14:37. doi: 10.1186/s13195-022-00979-9 (PMC8876266; doi:10.1186/s13195-022-00979-9)
Supplement: Supplementary file 1 — Additional file 1: Supplementary Table 1. Demographic and clinical data from the human brain tissue samples. [file 13195_2022_979_MOESM1_ESM.docx]

**Supplementary table 1: Demographic and clinical data from the human brain tissue samples**

| **No.** | **Sample Code ID** | **Specimens** | **Age** | **Sex** | **PMI(h)** | **Diagnosis** | **ApoE genotype** |
| --- | --- | --- | --- | --- | --- | --- | --- |
| **1** | 82 | Non-carrier | 69 |  | 22 | Normal | E3/E3 |
| **2** | 85 | Non-carrier | 71 |  | 12 | Normal | E3/E3 |
| **3** | 75 | Non-carrier | 56 |  | 19 | Normal | E3/E3 |
| **4** | 86 | Non-carrier | 82 |  | 24 | AD | E3/E3 |
| **5** | 173 | Non-carrier | 98 |  | 31 | AD | E3/E3 |
| **6** | 126 | Non-carrier | 75 |  | 24 | AD | E3/E3 |
| **7** | 96 | ApoE4 carrier | 89 | F | 34 | AD | E4/E4 |
| **8** | 182 | ApoE4 carrier | 74 | M | 12 | AD | E4/E4 |
| **9** | 192 | ApoE4 carrier | 83 | F | 8 | AD | E4/E4 |
|  |  |  |  |  |  |  |  |

**Supplementary Table 1.** Values are individual data points from each patient. Sex: M, Male. F, Female. Postmortem interval (h)l: time spam in hours from the moment the patient passed away to the moment the brain was collected. ApoE genotype: ApoE3/ApoE3 (E3/E3) and ApoE4/ApoE4 (E4/E4).
